# Vascular reaction parameters correlate with severe nerve fibre thickness loss and visual field defects in glaucoma

**DOI:** 10.1007/s10792-025-03810-0

**Published:** 2025-10-21

**Authors:** Julia Prinz, Matthias Fuest, Konstantin Kotliar, Peter Walter, Muriel Hollstein, Niklas Plange, David Kuerten

**Affiliations:** 1 Department of Ophthalmology, University Hospital RWTH Aachen, Pauwelsstr. 30, Aachen, Germany; 2 Department of Medical Engineering and Technomathematics, FH Aachen, Aachen, Germany

**Keywords:** Glaucoma, Ocular perfusion, RVA, Blood flow, Neurovascular coupling

## Abstract

**Purpose:**

Vascular risk factors and ocular perfusion abnormalities are key elements in the pathogenesis of glaucoma. The retinal vessel analyser (RVA; IMEDOS Systems, Germany) enables non-invasive assessment of dynamic changes in retinal vessel diameters in response to light stimulation. In this pilot study, we explored whether parameters of vascular regulation correlate with visual field defects and retinal nerve fibre layer (RNFL) thickness (SD-OCT, Spectralis) in glaucoma patients.

**Methods:**

A cross-sectional observational study was conducted involving 34 eyes from 34 patients with advanced visual field defects associated with primary open-angle glaucoma (POAG). Following pharmacological pupil dilation, RVA measurements were performed according to a standardised protocol including stimulation with monochromatic flicker light. The resulting vascular response parameters were then analysed for correlations with both global and hemispheric visual field defects, as well as global and corresponding hemispheric RNFL thickness.

**Results:**

Maximal venous dilatation (r = 0.39, *p* < 0.02) as well as arterial (r = 0.41, *p* < 0.01) and venous amplitude of vessel reaction (r = 0.31, *p* < 0.04) were significantly correlated to overall visual field defect mean deviation (MD). Venous maximal dilatation (r = 0.47, *p* < 0.004), the amplitude of vessel reaction (r = 0.37, *p* < 0.01) and the amplitude of arterial vessel reaction (r = 0.33, *p* < 0.02) were significantly correlated to overall RNFL thickness. Time until maximal dilatation in the arteries was significantly correlated to the corresponding hemispheric RNFL thickness (r = − 0.43, *p* < 0.007).

**Conclusion:**

Vascular reaction parameters show significant correlations with structural and functional impairment in advanced stages of glaucoma. These findings support the hypothesis that disturbed ocular blood flow and autoregulatory mechanisms may contribute to disease severity.

## Introduction

Glaucoma is a leading cause of blindness worldwide, currently affecting more than 60 million people [1, 2]. However, the pathogenesis and precise mechanisms underlying glaucomatous optic disc damage remain incompletely understood. Ocular hemodynamics and blood flow appear to play a crucial role in disease onset and progression, with evidence of vascular dysfunction observed in glaucoma patients [3–9]. A pathological ischemia–reperfusion injury cycle has been hypothesized, with particular emphasis on disruptions in the venous component of the ocular circulation [6]. Additionally, dysregulated neurovascular coupling is considered a key contributor to sustained ischemia–reperfusion injury [10].

The assessment and quantification of ocular blood flow remain challenging in both clinical practice and research. Traditional methods have failed to directly investigate neurovascular coupling, the mechanism by which blood flow is rapidly adjusted to meet the metabolic needs of active neural tissue [7]. Increased neural activity triggers neurotransmitter release from neurons, initiating a cascade that enhances blood flow to the active region. This process involves coordinated interactions among neurons, astrocytes, and endothelial cells in the neurovascular unit [7]. Dysregulation of this unit may contribute to vascular abnormalities linked to glaucoma [7]. In this context, the retinal vessel analyser (RVA, Imedos Systems, Jena, Germany) has emerged as a valuable tool for investigating ocular vascular dynamics, as demonstrated by numerous recent studies [11–13].

The RVA enables dynamic assessment of retinal blood flow changes in response to flicker light stimulation. Previous studies using this device have reported impaired vascular response patterns in patients with open-angle glaucoma [9, 10]. To date, no significant correlations have been established between recorded RVA parameters and glaucomatous damage.

Advanced glaucoma represents a particular clinical challenge, as structural–functional correlations become less consistent and many diagnostic parameters lose sensitivity in late disease stages [14]. In this context, identifying markers that remain reliable in advanced disease is of high clinical relevance. Therefore, this study specifically focused on patients with advanced glaucoma, aiming to evaluate whether the investigated parameters retain diagnostic and functional value in this subgroup.

In this cross-sectional observational study, we aimed to investigate the relationship between altered vascular reactivity and advanced structural and functional changes in patients with glaucoma. The mean deviation (MD) of the visual field, expressed in decibels (dB), was employed as the functional parameter, while retinal nerve fiber layer (RNFL) thickness, measured in micrometers, was used as the structural parameter in our analysis.

## Methods

A comprehensive medical history and ophthalmological examination were conducted for each patient prior to the measurements, including intraocular pressure (IOP) assessment with Goldmann applanation tonometry, achromatic visual field defect testing (Humphrey II, Zeiss, Germany), and measurement of RNFL thickness via spectral domain optical coherence tomography (SD-OCT, Heidelberg Engineering, Heidelberg, Germany) measurement. The nasal and temporal superior/inferior sectors were aggregated and an average for superior and inferior retinal nerve fibre thickness was calculated.

Diagnosis of primary open-angle glaucoma (POAG) was established based on thinning of the inferior or superior rim and/or a cup-to-disc ratio asymmetry greater than 0.2, not attributable to optic disc size asymmetry, in conjunction with compatible glaucomatous visual field deficits, defined as a cluster of three or more test points with greater than 5 dB sensitivity reduction or two points with greater than 10 dB reduction compared to age-corrected normal values, following European Glaucoma Society guidelines. Additionally, all patients underwent gonioscopic examination to confirm the presence of an open anterior chamber angle and to rule out secondary forms of glaucoma (e.g., pseudoexfoliative, pigmentary, or angle-closure mechanisms). IOP was documented to be ≥ 21 mmHg prior to the initiation of antiglaucomatous therapy.

All patients were receiving chronic topical antiglaucomatous therapy. To minimize acute pharmacological influences on vascular parameters, medication regimens were kept stable for at least four weeks prior to imaging; a complete medication withdrawal was not performed due to the advanced stage of glaucoma.

This cross-sectional observational study followed the tenets of the declaration of Helsinki. All study subjects gave their informed consent to participate in this study. The study was approved by the local ethic committee of the University Hospital RWTH Aachen (EK 293/16).

This study included 34 eyes of 34 patients (mean age 65.54 ± 13.44 years; 64.7% female, n = 22) diagnosed with POAG. Only patients with advanced glaucoma, defined as a MD worse than − 12 dB as well as advanced optic disc cupping (vertical cup to disc-ratio of at least 0.8) according to the European Glaucoma Society Guidelines [15], were included.

Exclusion criteria included ocular opacities that hindered the imaging of retinal vessels (e.g., severe cataract, vitreous opacities, corneal scarring). Additionally, patients with a history of epilepsy, angle-closure glaucoma, shallow anterior chamber with a risk of excessive IOP increase post-mydriasis, or any other glaucoma subtype aside from POAG were excluded. Other exclusion criteria encompassed an inability to undergo examinations, uncontrolled arterial hypertension, and the presence of any other ocular vascular diseases, such as diabetic retinopathy.

Topical anti-glaucomatous medications used included dorzolamide, latanoprost, timolol, brimonidine, and their combinations; no patients without anti-glaucomatous treatment were included in this study. All visual field examinations were conducted using the achromatic standard program of the Oculus Humphrey Visual Field Analyzer (24–2 SITA, Zeiss, Germany) prior to any RVA measurements, with the mean deviation (MD) calculated for the corresponding hemisphere.

Adequate pupil dilation was achieved with 2–3 drops of a tropicamide (Mydriaticum Stulln, Pharma Stulln GmbH, Stulln, Germany) administered 30 min prior to measurements. All assessments were performed between 1 and 4 PM to minimize the potential influence of diurnal variations.

### Retinal vessel analyser

The RVA, version DVAlight (dynamic vessel analyser, IMEDOS Systems, Jena, Germany) is a commercially available unit, consisting of a fundus camera (Zeiss, Germany), video capturing and recording device, and a real-time monitor connected to a personal computer with video processing software. The RVA and its technical features are presented in detail elsewhere [11, 16]. In short, vessel diameters of the retinal arterioles and venules are measured longitudinally at an observer-defined location of the fundus. Furthermore, the integration of a video recording software allows offline reassessment of the recorded data. The retinal vein and artery diameters are recorded precisely with temporal resolution of 25 readings per second. The retinal diameter is measured continuously. The predefined segments are chosen in the main vessel branches approximately one disc diameter away from the rim of the optic disc (ONH), to minimize disturbances due to blood flow turbulences. The patient is sitting upright with the head in a headrest and asked to look slightly up or down to a fixation needle depending on which vessels are captured. The fundus is illuminated with green light of a wavelength between 567 and 587 nm. Flicker stimulus is generated by interrupting the illuminating light with a frequency of 12.5 Hz, which is in the range of the optimal excitation frequency for retinal vessels in humans [8]. The flicker stimulation relies on the principle of neurovascular coupling [17, 18]. The device produces measurements with a high repeatability and reproducibility [8].

The standard measurement protocol consists of 3 episodes with 20 s flicker stimuli followed by 80 s of observation and took overall 350 s. The vessels in the superior and inferior halves of the retina were measured at a distance of 1–2 optic nerve head (ONH) diameters from the ONH rim.

For the statistical analysis, the hemisphere of the visual field was assigned to the corresponding retinal vessel reaction. Only measurements of sufficient quality were used for further analysis.

### Blood pressure and other biomedical parameters

Blood pressure and heart rate values were recorded before and after each measurement with the patient sitting upright.

### Data evaluation

The data was analysed separately using a template with corresponding macros in a spreadsheet (MS Excel, Microsoft, USA). With the template, each numerical data provided from the RVA was filtered, processed and analysed. For statistical purposes, absolute vessel diameters of the predetermined segments were calculated individually during the last 30 s before the first flickering [8]. It was measured in MU, where 1MU corresponds to 1 µm in the Gullstrand’s eye. The three individual response curves for each participant, consisting of a 30-s baseline assessment prior to flicker application, 20 s of flicker stimulation, and 80 s of post-flicker recovery, were averaged. The average temporal vessel response for each participant was calculated as the mean of these three curves. The absolute diameter recorded in the 30 s before the first flicker was set to 100% and all changes were recorded in % to the individual baseline value. For each patient, this normalized averaged time course of relative vessel diameter changes was smoothed using the running median (5 s frame) and the corresponding back shift. The following parameters of dynamic retinal vessel reaction were derived for each subject:

1. Mean maximal dilatation in response to flicker stimulation (absolute maximum of the smoothed normalized curve, (in % to the baseline)
2. Time until maximal vessel dilatation (in s)
3. Mean maximal constriction after flicker stimulation (absolute minimum of the smoothed normalized curve). For the curves under the 100%-line the value was negative; (in % to the baseline)
4. Time until maximal vessel constriction (in s)
5. Amplitude of the reaction (in % to baseline calculated as the difference between mean maximal dilation and constriction on the smoothed curve)

In short, the reaction patterns of the retinal arteries and veins show a bimodal reaction with 2 dilatation peaks during flicker light stimulation followed by a constriction after the flicker stimulation before returning to baseline diameter.

**Fig. 1:**
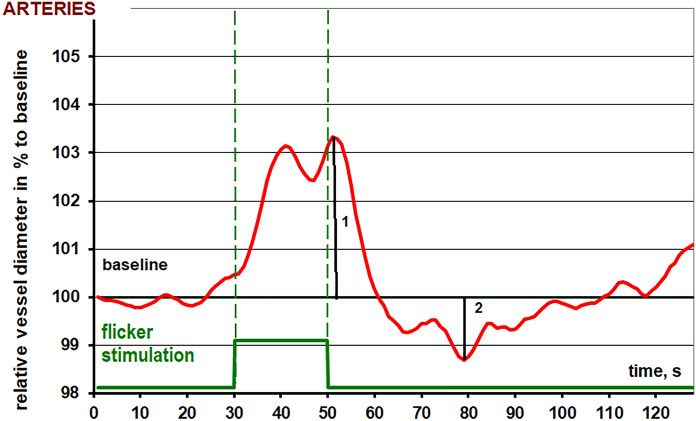
Exemplary arterial diameter response to flicker stimulation recorded by retinal vessel analysis (RVA)

An exemplary arterial reaction pattern recorded via RVA is presented in Fig. 1.

### Statistical analysis

Statistical analysis and graph generation were conducted using MS Excel (2000 for Windows) and GraphPad Prism (version 7.0 for Windows). Results were deemed statistically significant if the *p*-value was < 0.05. Descriptive statistics for non-normally distributed data are presented as the median and interquartile range (IQR, 1st–3rd quartile). Spearman correlation coefficients were calculated to assess relationships between variables.

## Results

The clinical parameters regarding systolic and diastolic blood pressure, IOP, visual field examination MD and average RNFL-thickness are provided in Table 1.

**Table 1:** Clinical parameters of the glaucoma patients

IOP (in mmHg)	hemispheric MD (in dB)	overall MD (in dB)	hemispheric average RNFL thickness (in µm)	general average RNFL thickness (in µm)	systolic blood pressure (in mmHg)	diastolic blood pressure (in mmHg)
14 (12–16)	− 18.75 (− 23.94/− 13.84)	− 13.7 (− 18.19/− 9.99)	55 (45/63.75)	50.5 (42.25/65.25)	156 (132/169)	81.5 (78/95)

The median and interquartile range (IQR), represented by the 1st (Q1) and 3rd (Q3) quartiles, are presented. *IOP* intraocular pressure, *MD* mean deviation, *RNFL* retinal nerve fiber layer

The correlation-coefficients as well as *p*-values for the correlation between RVA-parameters and hemispheric MD, overall MD and average as well as corresponding hemispheric RNFL thickness are provided in Tables 2, 3, 4 and 5.

**Table 2:** Correlation for arterial and venous vessel reaction parameters measured via retinal vessel analyser (RVA) and hemispheric visual field mean deviation (MD)

Correlation between hemispheric MD and	Arteries		Veins	
	r	*p*	r	*p*
Mean maximal dilatation	0.12	> 0.26	0.12	> 0.24
Time until maximal dilatation	− 0.12	> 0.26	− 0.1	> 0.29
Mean maximal constriction	0.09	> 0.4	− 0.05	> 0.38
Time until maximal constriction	0.07	> 0.34	− 0.23	> 0.1
Amplitude of the reaction	0.12	> 0.25	0.02	> 0.44
Vessel diameter	0.23	> 0.11	0.16	> 0.19

**Table 3:** Correlation for arterial and venous vessel reaction parameters measured via retinal vessel analyser (RVA) and overall visual field MD

Correlation between overall MD and	Arteries		Veins	
	r	*p*	r	*p*
Mean maximal dilatation	0.27	> 0.07	**0.39**	** < 0.02**
Time until maximal dilatation	− 0.08	> 0.32	− 0.07	> 0.35
Mean maximal constriction	− 0.27	> 0.06	− 0.17	> 0.18
Time until maximal constriction	− 0.05	> 0.39	− 0.27	> 0.07
Amplitude of the reaction	**0.41**	** < 0.01**	**0.31**	** < 0.04**
Vessel diameter	0.14	> 0.21	− 0.09	> 0.30

Statistically significant values (*p* < 0.05) are shown in bold

**Table 4:** Providing the correlation for arterial and venous vessel reaction parameters measured via retinal vessel analyser (RVA) and hemispheric average retinal nerve fiber layer (RNFL)-thickness (measured via spectral domain optical coherence tomography)

Correlation between hemispheric average RNFL thickness and	Arteries		Veins	
	r	*p*	r	*p*
Mean maximal dilatation	− 0.07	0.33	0.14	> 0.22
Time until maximal dilatation	− **0.43**	** < 0.007**	0.10	> 0.28
Mean maximal constriction	0.28	> 0.05	− 0.18	> 0.15
Time until maximal constriction	− 0.007	> 0.48	− 0.24	> 0.08
Amplitude of the reaction	− 0.10	> 0.28	0.14	> 0.22
Vessel diameter	0.26	> 0.07	0.004	> 0.49

Statistically significant values (*p* < 0.05) are shown in bold

**Table 5:** Correlation for arterial and venous vessel reaction parameters measured via retinal vessel analyser (RVA) and overall average retinal nerve fiber layer (RNFL)-thickness (measured via spectral domain optical coherence tomography)

Correlation between overall average RNFL thickness and	Arteries		Veins	
	r	*p*	r	*p*
mean maximal dilatation	0.17	> 0.17	**0.47**	** < 0.004**
time until maximal dilatation	0.001	> 0.49	− 0.1	> 0.28
mean maximal constriction	− 0.1	> 0.29	− 0.13	> 0.22
time until maximal constriction	− 0.09	> 0.29	− 0.24	> 0.09
amplitude of the reaction	**0.33**	** < 0.02**	**0.37**	** < 0.01**
vessel diameter	0.23	> 0.1	− 0.02	> 0.44

Statistically significant values (*p* < 0.05) are shown in bold

Venous dilatation, arterial and venous amplitude of vessel reaction were significantly correlated to overall visual field mean defect (r = 0.39, *p* < 0.02; r = 0.41, *p* < 0.01; r = 0.31, *p* < 0.04 respectively). Venous maximal dilatation and amplitude of vessel reaction were significantly correlated to overall RNFL thickness (r = 0.47, *p* < 0.004; r = 0.37, *p* < 0.01, respectively). For the arteries, time until maximal dilatation as well as amplitude of vessel reaction was significantly correlated to overall RNFL thickness (r = − 0.43, *p* < 0.007; r = 0.33, *p* < 0.02 respectively).

## Discussion

In this study, both arterial and venous reaction parameters correlated with overall mean visual field defect and RNFL thickness in patients with advanced stages of glaucoma. Higher vascular responses were recorded in less progressed glaucoma stages. Interestingly, beyond overall vessel dilatation parameters, we identified a significant correlation between an altered time-lapse response (i.e., time to peak dilatation) and RNFL thickness, where a delayed dilatation response was associated with lower RNFL thickness.

### Comparison with previous literature

Previous studies have reported impaired vascular dilatation in glaucoma patients. While most studies fail to demonstrate statistically significant alterations in arterial response parameters, venous changes have been consistently observed. Garhofer et al. reported a significantly reduced flicker-induced venous dilatation in 31 patients with early-stage POAG compared to healthy controls [12]. Similarly, Nagel et al. found that venous dilatation was significantly impaired in POAG patients following an artificial IOP increase, compared to both healthy controls and ocular hypertensive patients [19].

In our study, beyond venous reaction parameters, both the amplitude of arterial response and time to peak dilatation were significantly correlated with RNFL thickness and visual field mean defect. However, other arterial parameters, including mean maximal arterial dilatation and constriction, did not reach statistical significance (*p* > 0.07 and *p* > 0.06, respectively).

### Divergent evidence in early glaucoma studies

To date, no studies have reported significant correlations between RVA measurements and either functional or structural damage in early-stage glaucoma.

Gugleta et al. did not find significant correlations between RVA parameters and disease severity in 51 patients with POAG [20]. While baseline arterial diameter was initially correlated with average RNFL thickness, this association lost significance after multiple comparison correction. No correlations were identified between RVA parameters and visual field defects. Notably, the study included patients with relatively mild glaucomatous damage, with a MD of − 3.4 ± 3.2 dB in the "worse" eye, compared to − 13.91 ± 5.55 dB in our study cohort.

Similarly, Waldmann et al. did not observe significant correlations between the progression of visual field defects, retinal nerve fiber layer thinning, and maximal flicker-induced vascular responses in arteries and veins over a 3-year period [10].

Their prospective study included 56 POAG patients, 39 of whom demonstrated disease progression, while 17 remained stable. In contrast to our cohort, patients in the study by Waldmann et al. had less advanced disease, with a mean MD of − 4.1 ± 4.4 dB compared to − 13.91 ± 5.55 dB in our cohort. Additionally, their patients exhibited significantly higher RNFL values (72.9 ± 17.3 µm vs. 55.41 ± 16.99 µm in our study). Importantly, the 3-year observation period may have been insufficient to detect significant changes in a slowly progressive disease such as glaucoma, particularly in early stages. The authors themselves acknowledged that no significant worsening of visual field parameters was observed during the study period. Nevertheless, they reported that in eyes with intraocular differences, the hemisphere exhibiting a weaker flicker response was associated with greater RNFL thinning over time, suggesting a potential role of vascular dysregulation in retinal ganglion cell loss and the utility of RVA measurements in identifying patients at risk of progression.

However, previously published data from our group provide a different perspective. In patients with advanced altitudinal visual field defects, the hemisphere corresponding to the visual field loss exhibited greater vascular responses [21]. This suggests that vascular dysregulation may play a more prominent role in earlier glaucoma stages, while reduced vascular reactivity could precede glaucomatous damage. Whether this relationship is causal or merely coincidental remains uncertain.

Our study demonstrated significant associations between vascular reactivity, regulatory mechanisms, and both functional and structural disease parameters in advanced glaucoma. Based on previous publications, it is evident that these findings cannot be directly transferred to earlier stages of the disease.

### Hemispheric and localized vascular responses

Interestingly, we did not observe significant correlations between localized (i.e., hemispheric) visual field defects, RNFL thickness, and corresponding vascular responses. This suggests that neurovascular coupling may not be as strictly localized as previously assumed, or that an overall dysregulation of vascular function in glaucoma may obscure more specific regional associations. If lower RNFL values correspond to a reduced number of functioning neurons and axons, a delayed vascular response may be indicative of disrupted neurovascular coupling secondary to neuronal loss.

### The role of OCT-angiography for vascular assessment in glaucoma

The recently developed OCT-angiography (OCTA) has emerged as a valuable non-invasive tool for vascular research in ophthalmology. An increasing body of evidence suggests that reduced vessel density and vascular rarefaction are associated with glaucoma severity and can be quantitatively correlated with disease progression [22]. A key algorithm for detecting ocular perfusion in small peripapillary vessel branches, known as split-spectrum amplitude-decorrelation angiography (SSADA), was introduced by Jia et al. in 2012 [23].

Wang et al. reported that in 62 glaucomatous eyes, subdivided into three groups based on the extent of visual field impairment, both the peripapillary flow index and vessel density correlated with MD and RNFL thickness [24]. These findings align with previously reported changes observed using other imaging techniques, such as fluorescein angiography [25] or Colour Doppler Imaging (CDI) [26], which have demonstrated reduced blood flow in regions of advanced glaucomatous damage.

Despite its advantages, OCT-angiography has certain limitations. It does not allow for continuous assessment of ocular blood flow or vascular regulation. Additionally, OCT signals from major blood vessels may be absent due to the washout of interferometric fringes within the OCT signal integration time [27]. Nevertheless, a major advantage of OCT-angiography is its excellent reproducibility within and between visits, as well as its strong inter-operator reliability [28]. The same authors have suggested—and we fully agree—that perfusion indices may serve as more valuable indicators for glaucoma staging and monitoring than structural measurements [21], underscoring the importance of reliable ocular blood flow assessments in future research.

### Study limitations

Several limitations of our study should be acknowledged. First, the cross-sectional design does not allow for conclusions regarding causality or temporal relationships. The observed associations should therefore be interpreted with caution. Second, the relatively small sample size (34 eyes) limits the statistical power and generalizability of the findings. The study population represents a selected subgroup of patients with advanced glaucoma who agreed to undergo time-consuming, detailed examinations, potentially introducing selection bias. As such, the results should be considered exploratory. Larger, longitudinal studies are needed to confirm these preliminary findings and to further investigate the clinical relevance of the observed associations. Further, the RVA allows for the assessment of vascular dynamics in superficial retinal vessels originating from the central retinal artery (CRA). However, only the superficial layers of the ONH are directly supplied by CRA branches, while the deeper layers receive their blood supply primarily from the posterior ciliary arteries (PCAs) [29]. Given that glaucomatous ONH damage is thought to originate in regions nourished by the PCAs [30], RVA measurements do not provide a direct assessment of ONH perfusion. Instead, our findings may reflect overall vascular dysregulation or, more specifically, impaired neurovascular coupling in glaucomatous eyes. Notably, reduced flow velocities in the CRA have been consistently observed in glaucoma patients using CDI [26, 31], and we previously identified a correlation between peak systolic velocity (PSV) in the CRA and visual field progression in NTG patients [32].

Furthermore, RVA measurements require a high level of patient cooperation. While good reproducibility has been reported [11], the technique remains challenging for both investigators and patients. Additionally, our study primarily included patients with advanced glaucoma, and the findings may not be generalizable to all disease stages.

Finally, individual topical medication as well as different systemic medication was taken at the time of the measurements, which may have interfered with the vascular reaction and subsequent RVA measurements. Only one report regarding the effect of topical antiglaucomatous medication on vascular reaction detected measured via RVA is published to date showing that overall vessel dilatation is not impaired by topical dorzolamide, whereas the overall reaction seems to be faster in glaucomatous eyes treated with dorzolamide after artificial IOP rise [33]. In this study, all patients were under chronic topical therapy with stable medication regimens maintained for at least four weeks prior to imaging, reducing the likelihood of acute pharmacological effects. However, the long-term impact of these agents on retinal perfusion remains insufficiently studied. Some authors found timolol to increase ocular blood flow in glaucomatous patients [34], whereas others found that ocular blood flow was decreased [35]. Similar results were reported for topical dorzolamide. Some authors found improved ocular blood flow parameters [34], whereas other authors did not find any changes [36]. Due to the cross-sectional and exploratory nature of the study, medication-specific stratification was not feasible. Future investigations specifically designed to isolate and control for these effects are needed to better understand the role of topical therapy in retinal vascular regulation.

## Conclusion

This cross-sectional observational study demonstrates, for the first time, that impaired vascular responses are associated with the extent of structural and functional damage in advanced stages of glaucoma. Future large-scale studies, encompassing different glaucoma stages and accounting for the influence of topical medication, are necessary to further evaluate the potential of RVA measurements as a valuable tool in identifying patients at risk of irreversible glaucomatous disease progression.

## Data Availability

No datasets were generated or analysed during the current study.
